# Prolonged ICU Stay in Severe and Critically-Ill COVID-19 Patients Who Received Convalescent Plasma Therapy

**DOI:** 10.1155/2022/1594342

**Published:** 2022-09-07

**Authors:** Bambang Pujo Semedi, Nadya Noor Ramadhania, Betty Agustina Tambunan, Siprianus Ugroseno Yudho Bintoro, Soedarsono Soedarsono, Cita Rosita Sigit Prakoeswa

**Affiliations:** ^1^Anesthesiology and Reanimation Department, Faculty of Medicine, Airlangga University-Dr. Soetomo General Academic Hospital, Surabaya, Indonesia; ^2^Clinical Pathology Department, Faculty of Medicine, Airlangga University-Dr. Soetomo General Academic Hospital, Surabaya, Indonesia; ^3^Internal Medicine Department, Faculty of Medicine, Airlangga University-Dr. Soetomo General Academic Hospital, Surabaya, Indonesia; ^4^Pulmonology and Respiratory Medicine Department, Faculty of Medicine, Airlangga University-Dr. Soetomo General Academic Hospital, Surabaya, Indonesia; ^5^Faculty of Medicine, Airlangga University-Dr. Soetomo General Academic Hospital, Surabaya, Indonesia

## Abstract

**Background:**

Convalescent plasma administration in severe and critically-ill COVID-19 patients have been proven to not provide improvement in patients' outcome, yet it is still widely used in countries with limited resources due to its high availability and safety. This study aims to investigate its effects on ICU mortality, ICU length of stay (LoS), and improvement of oxygen support requirements.

**Methods:**

Data of all severe and critically-ill patients in our COVID-19 ICU was collected retrospectively between May and November 2020. We dichotomized the variables and compared outcome data of 48 patients, who received convalescent plasma to 131 patients, receiving standard of care. Data were analyzed using multiple logistic regression to make prediction models of mortality, length of stay, and oxygen support device requirement.

**Result:**

Overall mortality rate in our COVID-19 ICU was 55.3%, with a median overall length of stay of 8 (4–11) days. Less patients that received convalescent plasma presented with the need for mechanical ventilation on ICU admission (*p* < 0.001), but with comparable PaO_2_ to FiO_2_ (P/F) ratio (*p*=0.95). Factors that confounded mortality were obesity (aOR = 14.1; 95% CI (1.25, 166.7); *p*=0.032), mechanical ventilation (aOR = 333; 95% CI (4.5,1,000); *p* < 0.001), higher neutrophil-to-lymphocyte ratio (NLR) (aOR = 7.32; 95% CI (1.82, 29.4); *p*=0.005), and lower P/F ratio (aOR = 7.70; 95% CI (2.04, 29.4); *p*=0.003). ICU LoS was longer in patients, who had prior history of hypertension (aOR = 2.14; 95% CI (1.05, 4.35); *p*=0.036) and received convalescent plasma (aOR = 3.88; 95% CI (1.77, 8.05); *p* < 0.001). Deceased patients, who received convalescent plasma, stayed longer in the ICU with a mean length of stay of 12.87 ± 5.7 days versus 8.13 ± 4.8 days with a significant difference (*U* = 434; *p* < 0.000). The chance of improved oxygen support requirements was lower in obese patients (aOR = 9.18; 95%CI (2.0, 42.1); *p* < 0.004), mechanically ventilated patients (aOR = 13.15; 95% CI (3.75, 46.09); *p* < 0.001), patients with higher NLR (aOR = 2.5; 95% CI (1.07, 5.85); *p*=0.034), and lower P/F ratio (aOR = 2.76; 95% CI (1.1, 6.91); *p*=0.031).

**Conclusion:**

The length of stay of patients in the convalescent plasma group was significantly longer than the control group. There was no effect of convalescent plasma in ICU mortality and no improvement was observed in terms of oxygen support requirements.

## 1. Introduction

The Coronavirus disease 2019(COVID-19) pandemic has significantly affected healthcare worldwide. The disease is associated with rapid virus spread, a high surge of cases, followed by large numbers of critically-ill patients with respiratory failure in need of intensive care unit (ICU) beds and mechanical ventilation. Exponential increase of cases in a short time had put a huge strain on healthcare facilities. In the beginning of the COVID-19 pandemic, Indonesia was ranked as one of the countries with the highest COVID-19 mortality rate, with East Java province being the highest contributor [1, 2]. Researchers and clinicians worldwide were in search of potential treatments to aid the recovery of COVID-19 patients. One of the proposed treatment was convalescent plasma due to its history of previous viral infections, while also being an affordable and widely-available treatment choice in developing countries.

Convalescent plasma had proven its benefits in the treatment of Severe Acute Respiratory Syndrome Coronavirus (SARS-CoV) [3, 4], Middle East Respiratory Syndrome Coronavirus (MERS-CoV) [5], H1N1 influenza [6], and H5N1 influenza infections [7]; thus, it was proposed as a potential treatment for COVID-19. One of the first convalescent plasma study conducted in China on 10 severely ill patients showed marked clinical improvement, decreased viral load, and good outcomes [8]. Another study conducted in Italy at the beginning of the COVID-19 pandemic on 46 COVID-19 patients with severe pneumonia showed that convalescent plasma transfusion reduced mortality, decreased inflammatory markers, and showed improvement in respiratory function [9]. Other studies also showed favorable outcomes [10, 11]. However, large sample trials had shown no difference in terms of these parameters after convalescent plasma administration. One of them is a randomized, controlled, open-label, platform trial known as the recovery trial. The trial was conducted in 177 NHS hospitals in the UK with 11,558 COVID-19 patients enrolled (5,975 patients in plasma group and 5,763 patients in control group). No difference was observed in terms of outcomes between the groups, despite the high titer of plasma used in the study [12].

Several studies were conducted to assess the safety of this treatment modality. A study in 25 severely ill COVID-19 patients who received convalescent plasma proved its safety, with no obvious adverse effect related to plasma administration reported [13]. Another early study in China reported no plasma-related severe adverse events [8]. However, a larger trial in China with 52 patients receiving convalescent plasma reported two adverse events, one mild nonsevere allergic transfusion reaction and the other severe transfusion-associated dyspnea. Both patients recovered with supportive care and medications [14].

The Indonesian Food and Drug Authority (FDA) released a recommendation for the use of convalescent plasma on May 15^th^, 2020 [15]. Since then, convalescent plasma had been widely used throughout the country on COVID-19 patients, mostly on those, who contracted severe and life-threatening COVID-19 in the ICU setting and moderately ill patients, who are at high risk of worsening conditions under close observation. In January 2021, the Indonesian Red Cross, in collaboration with the Indonesian government, Indonesian FDA, and National Disaster Management Authority declared a national initiative on convalescent plasma donors, encouraging COVID-19 survivors to be plasma donors [16].

Several trials with large sample sizes had been published earlier that showed no improvement after the administration of convalescent plasma. However, it is still frequently used in countries with limited resources due to its high availability and safety. We aim to find the benefits and disadvantages of convalescent plasma in the ICU setting in a country with limited access to resources.

## 2. Material and Methods

### 2.1. Study Design and Subjects

This study is a retrospective study at Dr. Soetomo General Hospital, Surabaya, Indonesia. The population of this study was 179 severe and critically-ill patients admitted to and discharged from our COVID-19 ICU between 1 May 2020 and 30 November 2020. Data were obtained from daily morning reports of COVID-19 ICU patients.

The inclusion criteria of plasma convalescent recipients were patients, who tested positive using RT-PCR for SARS-CoV-2 on samples taken from nasopharyngeal and oropharyngeal swabs, aged 18 years old or older, male or nonpregnant female, and received 300 ml of convalescent plasma twice during ICU hospitalization. Severe illness was defined as patients with dyspnea, respiratory rate of more than 29 times per minute, oxygen saturation of less than 93% measured in room air, or those with worsened radiographic findings of more than 50% in less than 48 hours. Critical illness was defined as patients with severe, progressive pneumonia that did not respond to standard treatment, acute respiratory distress syndrome (ARDS) confirmed by blood gas analysis PaO_2_/FiO_2_ value of less than 300, patients who received mechanical ventilation, and patients, who fell into septic shock and organ failure. The exclusion criteria were patients with end-stage renal disease who required regular hemodialysis, and patients with a history of severe allergic reaction to blood transfusion.

The control group was all patients, who were admitted to our COVID-19 ICU during the study period, and who did not receive convalescent plasma during hospitalization. We excluded patients who were admitted for postoperative observations, postpartum women, and patients with end-stage renal disease who required regular hemodialysis. Differences in baseline variables between both groups were compared using univariate analyses, categorical variables were tested with chi-square test, and continuous variables were tested using either an independent *t*-test or Mann‒Whitney test.

The power of this study was greater than 95% using previous study conducted on patients with severe and critical COVID-19 as reference with 13% mortality in convalescent plasma group compared to our data of 57% overall ICU mortality [10, 17].

### 2.2. Convalescent Plasma

Donors were recruited using the following inclusion criteria: male or nonpregnant females aged 18 to 60 years old; recovered from COVID-19 and confirmed negative for SARS-CoV-2 using RT-PCR on samples taken from nasopharyngeal and oropharyngeal swabs; symptom-free for at least 14 days before plasma donation; and did not have any comorbidities such as insulin-dependent diabetes mellitus, uncontrolled hypertension, chronic kidney disease and difficult vascular access. Donors were tested for serum-specific SARS-CoV-2 antibody and only the ones who had more than 1 : 320 titers continued to the plasmapheresis procedure. Contact shock freezer was used to freeze blood bags containing plasma and stored in a freezer under temperatures between −20 and −32 degrees Celsius.

### 2.3. Standard of Care

Patients suspected and confirmed for COVID-19 were all admitted into isolation wards. Those who declined to severe and critical conditions were treated in an ICU specifically dedicated to COVID-19 patients. Patients were given supportive therapy, such as oxygen, fluid, and nutrition therapy, along with supportive medications such as multivitamins, immuno-modulatory agents, anticoagulants, antioxidants, and corticosteroids. This study was conducted in the early phase of COVID-19 pandemic when drug availability varied worldwide. Therefore, the administration of antivirals at the time was dependent on the availability of the agent. Antibiotics and/or antifungals were given if there was any suspicion of bacterial and or fungal infections. Antibiotics were given empirically before culture-sensitivity test results were retrieved. Oxygen therapy was given to treat hypoxemia and to prevent organ failure due to respiratory distress. Patients who received oxygen therapy were those whose oxygen saturation levels were ≤93%. Oxygen support therapy was titrated from nasal cannula, face mask, high flow nasal cannula, noninvasive ventilation, and mechanical ventilation. Fluid therapy was given to patients for resuscitation and maintenance purposes. We used crystalloid for resuscitation fluid, preferably a balanced solution to prevent acid-base disturbances. We evaluated the patients' fluid responsiveness using dynamic parameters as a guide, preferring a conservative strategy to liberal. Maintenance fluid was adjusted to patients' needs and output, taking volume, electrolytes, and calories into account. Vasopressor was given if patients' improvement after adequate fluid administration was not sufficient. First choice of vasopressor was norepinephrine, with a target mean arterial pressure of 65 mmHg.

### 2.4. Measures

Outcomes measured in this study were ICU mortality, ICU length of stay (LoS), and improvement of oxygen support requirements. ICU LoS was the number of treatment days in the ICU and categorized into less than 7 days and 7 days or more, while improvement of oxygen support requirements refers to the type of oxygen support needed by the patient at ICU discharge compared to the initial support device needed at ICU admission. Baseline characteristics of patients were recorded, including gender, age, diagnostic status, duration from onset of symptoms to ICU admission, initial presenting symptoms, mechanical ventilation on admission, whether or not a patient was a healthcare worker, known contact with a confirmed case, comorbidities such as hypertension, diabetes mellitus, and obesity, laboratory findings such as white blood cells count, neutrophil-lymphocyte ratio, PaO_2_/FiO_2_ ratio, D-dimer, serum creatinine, and random blood glucose on admission.

### 2.5. Statistical Analysis

Categorical variables were presented as percentages, while continuous variables were presented as mean and standard deviation for variables with normal distribution and as median and interquartile range (IQR) for variables with skewed distribution.

We categorized all continuous variables into dichotomous variables and analyzed the relationships with outcome measures between plasma and control group using chi-square test. Dichotomization was based either on normal value or median. The results were expressed as odds ratio (OR), 95% confidence interval (CI), and *p*-value. Results were significant if *p*-value was less than 0.05. Variables with a *p*-value of less than 0.2 in the chi-square analysis were included in the further multivariate logistic regression analysis. The results were expressed as adjusted odds ratio (aOR), 95% confidence interval (CI), and *p*-value. Results were significant if *p*-value were less than 0.05. We did post-hoc analysis of ICU LoS using Mann‒Whitney U nonparametric test, to compare LoS between two groups. We conducted all of the statistical analysis using SPSS software.

## 3. Results

### 3.1. Patients' Characteristics

This study included 179 critically-ill patients hospitalized in our COVID-19 ICU from May to November 2020. We excluded 65 patients, who did not meet our inclusion criteria. The mortality within our sample was 55.3% (*n* = 99). The percentage of male patients was larger in both groups. Mean age of patients in both groups were around 50 years old (51.2 ± 11.6 and 49.5 ± 11.4 in the control and CP groups, respectively). Around 80% of patients were admitted to the ICU with confirmed COVID-19 cases, while others were still suspected of COVID-19 with unreleased RT-PCR for SARS-CoV2 results. Almost half of the patients were healthcare workers (doctors, nurses, dentists, radiographers, and transporters). Most patients experienced fever and mild respiratory symptoms (e.g., sore throat, cough, stuffy and runny nose) as first recognized symptoms. Most patients did not know who they came in contact with. Around half of the patients, who did not receive CP were intubated on admission, but 78.1% of patients who received CP did not receive mechanical ventilation on their first day in the COVID-19 ICU.

Fewer patients that received convalescent plasma presented with the need for mechanical ventilation on ICU admission (*p* < 0.001), but with comparable PaO_2_ to FiO_2_ (P/F) ratio (*p*=0.95). Initial white blood cells count (*p* < 0.001), D-dimer levels (*p* < 0.001), and creatinine serum (*p*=0.03) were lower in CP group. Baseline characteristics are shown in Table 1.

### 3.2. Outcome Parameters

In bivariate analysis, factors that confounded mortality were age group of more than 45 years old, mechanical ventilation both on admission and during ICU stay, baseline laboratory parameters such as higher white blood cells count, higher neutrophil-to-lymphocyte ratio, lower PaO_2_ to FiO_2_ ratio, higher D-dimer, higher creatinine serum, and higher random blood glucose levels. Factors that affected ICU length of stay were convalescent plasma administration, history of hypertension, higher WBC count, and lower PaO_2_ to FiO_2_ ratio. Oxygen support device improvement was related to obesity, mechanical ventilation on admission and during ICU stay, lower PaO_2_ to FiO_2_ ratio, and higher creatinine serum. We later made multivariate models of these outcome parameters. Bivariate analysis of variables is shown in Table 2.

**Figure 1:**
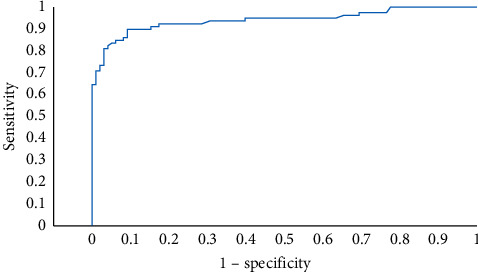
Receiver operating characteristics graph of mortality prediction model.

Multivariate analysis of mortality found that obese patients were more prone to death (aOR = 14.1; 95%CI [1.25, 166.7]; *p*=0.032). Patients, who received mechanical ventilation during their ICU stay also had a significantly higher risk of mortality by 333 times (aOR = 333; 95%CI (4.5, 1,000); *p* < 0.001). Higher neutrophil-to-lymphocyte ratio (aOR = 7.32; 95%CI (1.82, 29.4); *p*=0.005) and lower PaO_2_ to FiO_2_ ratio (aOR 7.70; 95%CI (2.04, 29.4); *p*=0.003) were also predictors of mortality in the COVID-19 ICU (Table 3). The area under ROC curve of this mortality prediction model was 0.949 (95%CI (0.914, 0.985); *p* < 0.001) (Figure 1).

**Figure 2:**
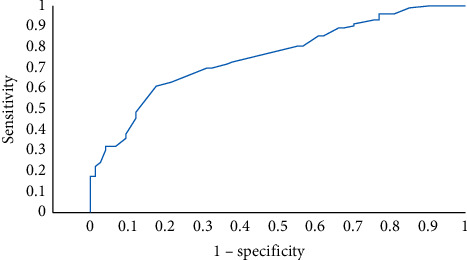
Receiver operating characteristics graph of ICU length of stay prediction model.

ICU length of stay was longer in patients, who had a prior history of hypertension (aOR = 2.14; 95%CI (1.05,4.35); *p*=0.036) and those, who received convalescent plasma (aOR = 3.88; 95%CI (1.77, 8.55); *p*=0.001), as shown in Table 4. The area under ROC curve of this model was 0.725 (95%CI (0.651, 0.799); *p* < 0.001) (Figure 2).

**Figure 3:**
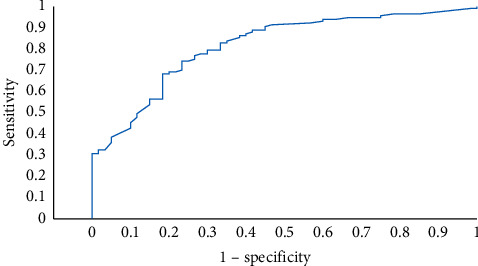
Receiver operating characteristics graph of constant and deteriorating oxygen support device.

The chance of constant and deteriorated oxygen support device was higher in obese patients (aOR = 9.18; 95%CI (2.0, 42.1); *p*=0.004), those who were intubated during ICU stay (aOR = 13.15; 95%CI (3.75, 47.09); *p* < 0.001), patients with higher neutrophil-to-lymphocyte ratio (aOR = 2.5; 95%CI (1.07, 5.85); *p*=0.034) and lower PaO_2_ to FiO_2_ ratio (aOR 2.76; 95%CI (1.1, 6.91); *p*=0.031), as shown in Table 5. This model had area under ROC curve of 0.819 (95%CI (0.753, 0.884); *p* < 0.001) (Figure 3).

**Figure 4:**
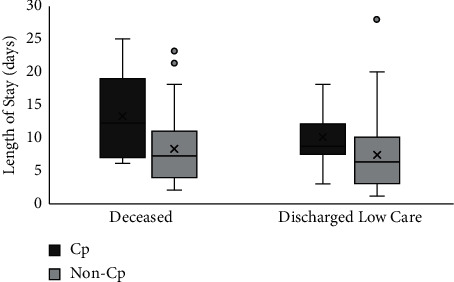
ICU length of stay of both groups was grouped by outcome.

We did a post-hoc analysis for duration of stay of patients in ICU, we separated those who survived and those who died during ICU stay, as pictured in Figure 4. We found that deceased patients who received convalescent plasma stayed longer in the ICU with median LoS of eleven (IQR = 5) days versus seven (IQR = 5) days with significant difference (*U* = 434; *p* < 0.000). Patients, whose condition improved and discharged to lower level of care also showed a similar results with patients receiving convalescent plasma who stayed longer in the ICU, with median LoS of nine (IQR = 4.5) days versus six (IQR = 7) days with significant difference (*U* = 400; *p*=0.004).

### 3.3. Convalescent Plasma Recipient

In this study, 48 patients received convalescent plasma transfusion. Only one patient received plasma within 3 days from onset. The mean duration of CP administration was 11.3 ± 4.57 days after onset of disease. None of these patients experienced severe adverse reactions. Although most patients did not receive mechanical ventilation on their first day in the ICU, 29 patients (60.4%) were later intubated during their ICU stay. Convalescent plasma was not related to mortality (*p*=0.22) nor oxygen support improvement (*p*=0.08), but was associated with significantly longer ICU stay (*p* < 0.001).

## 4. Discussion

Convalescent plasma had been used as one of the treatment choices for critical COVID-19 patients in Indonesia in early 2020, due to its promising history of benefits in other coronavirus pandemic and its high availability in the country. After more than one year, several studies with large sample sizes had been conducted and revealed that convalescent plasma mostly showed no significant benefit in both severe and critically-ill COVID-19 patients. However, convalescent plasma was still commonly used in the intensive care setting in Indonesia, as it remains one of the treatment options with the highest availability throughout the country.

This pandemic increased the demand of ICU beds and ventilators in such a short time. Treatments that could lead to faster recovery was crucial to increase ICU bed availability and reduce patients' treatment cost. Although a study had demonstrated a favorable outcome of convalescent plasma to reduce patients' length of stay [18], most studies showed no improvement in time to clinical improvement [14, 19, 20]. However, our study showed that patients, who received convalescent plasma stayed longer in the ICU (aOR = 3.88; *p*=0.001). Deceased patients, who received convalescent plasma had prolonged ICU stay with a mean duration of 12.87 ± 5.7 days versus 8.13 ± 4.8 days in the control group, indicating that the use of convalescent plasma in critically-ill patients could possibly increase the cost of treatment and did not lead to a good outcome.

Our center is the highest referral hospital in East Java, and most of our patients had been treated previously and referred from other hospitals throughout the province. The median number of days of ICU admission was 8 days after onset, and almost half of these patients presented in our ICU in life-threatening conditions requiring mechanical ventilation. The result of this study is likely due to the late administration of convalescent plasma. The mean number of days of convalescent plasma administration in our study was 11 days after onset. According to a multicenter study in the US, the 7-day and 30-day mortality rate of patients who received convalescent plasma at least 4 days after diagnosis were significantly higher than those who received it within 3 days after diagnosis (11.9% vs. 8.7%; *p* < 0.0001) [21]. Another study in the USA included 38 patients receiving convalescent plasma and showed that severely-ill patients who received such treatment showed significantly lower mortality (13% vs. 55%; *p* < 0.02) and shorter duration of stay (15.4 vs. 33; *p* < 0.01) compared to critically-ill patients [22].

Since the convalescent plasma was administered late into the course of disease, the patients might have already developed high titers of antibodies, resulting in no effect of plasma administration. A convalescent plasma clinical trial in severe COVID-19 pneumonia patients in the Netherlands was halted prematurely since most of the patients already had COVID-19 antibodies at baseline that were comparable to the antibody detected in the donors (1 : 160 vs. 1 : 160; *p*=0.40). The median of convalescent plasma administration in this study was 10 (IQR 6–15) days after onset, similar to ours. This study also showed no difference in Day-15 disease severity and mortality between both groups [20]. As older studies about CP in previous viral infections had stated, CP was more effective if administered early in the course of the disease, before the peak viremia, where massive IgM and IgG antibodies were produced. [23, 24]. A serial plasma study of COVID-19 patients showed that less than 40% of patients developed antibodies within the first week of the disease, and rapidly increased to 100% after the second week [7]. Highest point of viremia in viral infections typically occurs between 10 and 14 days, therefore, CP should be most effective if administered within the first week after the onset of symptoms. [1, 25].

We also produced three prediction models of outcomes of critically-ill COVID-19 patients in our ICU using standard clinical and laboratory parameters that are mostly available even in remote areas with limited diagnostic facilities. This information can be used as one of educational materials for patients and their families to predict patients' prognoses.

### 4.1. Limitations

Our hospital is the top referral hospital in our province, hence patients in our care had received treatments from other facilities and presented to us later in the course of their disease. Patients in the convalescent plasma group had a better initial assessment since the patients with better prognoses were more prioritized for convalescent plasma due to its limited availability. We did not have data on baseline antibody titers of the patients and the plasma products.

## 5. Conclusion

Convalescent plasma showed no effect in reducing ICU mortality and in improving oxygen support requirements. However, this study showed that convalescent plasma significantly lengthened patients' length of stay in the ICU.

## Figures and Tables

**Table 1:** Baseline characteristics of both groups.

Characteristics	Control *n* = 131	Plasma *n* = 48	*p*
Gender-*n* (%)			
Male	88 (67.2)	36 (75)	0.32
Female	43 (32.8)	12 (25)	

Age			
Mean (SD)	51.2 (11.6)	49.5 (11.4)	0.36

Age group-*n* (%)			
< 45 years	35 (26.7)	16 (33.3)	0.39
> 45 years	96 (73.3)	32 (66.7)	

Status-*n* (%)			
Confirmed infection	106 (80.9)	37 (77.1)	0.57
Suspected infection	25 (19.1)	11 (22.9)	

Healthcare worker-*n* (%)			
Yes	61 (46.6)	23 (47.9)	0.87
No	70 (53.4)	25 (52.1)	

Duration from onset of symptoms to ICU admission-days			
Median (IQR)	8 (6)	8 (3)	0.43

Known contact with confirmed-case-*n* (%)			
Yes	17 (13)	12 (25)	0.06
No	114 (87)	36 (75)	

Known comorbidities			

Hypertension-*n* (%)			
Yes	43 (32.8)	15 (31.3)	0.84
No	88 (67.2)	33 (68.8)	

Diabetes mellitus-*n* (%)			
Yes	61 (46.6)	15 (31.3)	0.07
No	70 (53.4)	33 (68.8)	

Obesity-*n* (%)			
Yes	18 (13.7)	7 (14.6)	0.89
No	113 (86.3)	41 (85.4)	

Initial symptoms			

Fever-*n* (%)			
Yes	69 (52.7)	30 (62.5)	0.67
No	62 (47.3)	18 (37.5)	

Mild respiratory symptoms-*n* (%)			
Yes	72 (55)	29 (60.4)	0.51
No	59 (45)	19 (39.6)	

Shortness of breath-*n* (%)			
Yes	35 (26.7)	13 (27.1)	0.96
No	96 (73.3)	35 (72.9)	

Gastrointestinal symptoms-*n* (%)			
Yes	16 (12.2)	2 (4.2)	0.13
No	115 (87.8)	46 (95.8)	

Mechanical ventilation			

Mechanical ventilation on admission-*n* (%)			
Yes	68 (51.9)	11 (22.9)	<0.01
No	63 (48.1)	37 (78.1)	

Mechanical ventilation during ICU stay-*n* (%)			
Yes	84 (64.11)	29 (60.4)	0.65
No	47 (35.9)	19 (39.6)	

Laboratory findings			

White blood cells count (×10^9^/L)			
Mean (SD)	12.6 (6)	9.12 (4.3)	<0.001

Neutrophil-Lymphocyte ratio			
Median (IQR)	11.2 (12.25)	9.2 (10.8)	0.21

PaO_2_/FiO_2_ Ratio			
Median (IQR)	110 (114,9)	117.4 (68.3)	0.95

D-dimer (ng/mL)			
Median (IQR)	2,130 (7,730)^1^	1,100 (1,747.5)	<0.001

Creatinine serum (mg/dL)			
Median (IQR)	1.0 (0.6)	0.9 (0.4)	0.03

Random blood glucose (mg/dL)			
Median (IQR)	177 (113)	145.5 (128.5)	0.41

Outcome measures			

Mortality-*n* (%)			
Deceased	76 (58)	23 (47.9)	0.23
Discharged to low-care	55 (42)	25 (52.1)	

Length of stay			
Mean (SD)	7.74 (5.02)	11.27 (4.97)	<0.001

Length of stay-*n* (%)			
<7 days	68 (51.9)	7 (14.6)	<0.001
>7 days	63 (48.1)	41 (85.4)	

Improvement of oxygen support device-*n* (%)			
Yes	40 (30.5)	20 (41.7)	0.16
No	91 (69.5)	28 (58.3)	

^1^Baseline D-dimer was measured in 48 (100%) patients in plasma group and 122 (93%) patients in control group.

**Table 2:** Bivariate analysis of variables.

Variable	Deceased	*p*	LOS <7 days	*p*	No O_2_ support device improvement	*p*
Convalescent plasma-*n* (%)						
Yes	23 (47.9)	0.23	12 (25.0)	<0.001	20 (41.6)	0.16
No	76 (58.0)		76 (58.0)		40 (30.5)	

Age group-*n* (%)						
<45 years	22 (43.1)	0.04	29 (56.8)	0.194	18 (35.3)	0.75
>45 years	77 (60.2)		59 (46.1)		42 (32.8)	

Hypertension-*n* (%)						
Yes	38 (65.5)	0.058	21 (36.2)	0.017	21 (36.2)	0.598
No	61 (50.4)		67 (55.3)		39 (32.2)	

Diabetes mellitus-*n* (%)						
Yes	44 (57.9)	0.549	35 (46.0)	0.475	26 (34.2)	0.398
No	55 (53.4)		53 (51.4)		34 (33.0)	

Obesity-*n* (%)						
Yes	17 (68.0)	0.173	13 (52.0)	0.759	3 (12.0)	0.02
No	82 (53.2)		75 (48.7)		57 (37.0)	

Mechanical ventilation on admission-*n* (%)						
Yes	67 (84.8)	<0.001	39 (49.3)	0.96	16 (20.2)	0.001
No	63 (4.5)		49 (49.0)		44 (44.0)	

Mechanical ventilation during ICU stay-*n* (%)						
Yes	96 (84.9)	<0.001	51 (44.0)	0.159	20 (17.7)	<0.001
No	47 (35.9)		37 (56.0)		40 (60.6)	

WBC^1^ count >11 × 10^9^/L-*n* (%)						
Yes	57 (66.2)	0.005	49 (57.0)	0.045	25 (29.0)	0.23
No	42 (45.1)		39 (41.9)		35 (37.6)	

Neutrophil-Lymphocyte ratio >11-*n* (%)						
Yes	61 (67.7)	<0.001	46 (51.1)	0.6	23 (25.5)	0.08
No	38 (42.7)		42 (47.2)		37 (41.5)	

PaO_2_/FiO_2_ ratio <200-*n* (%)						
Yes	89 (61.8)	<0.001	65 (45.1)	0.031	43 (29.8)	0.038
No	10 (28.5)		23 (65.7)		17 (48.6)	

D-dimer (ng/mL) >1000 ng/mL-*n* (%)^2^						
Yes	74 (62.7)	0.006	61 (51.7)	0.35	35 (29.7)	0.13
No	25 (40.9)		27 (44.3)		25 (50.0)	

Creatinine serum >1.2 mg/dL-*n* (%)						
Yes	48 (76.1)	<0.001	31 (49.2)	0.99	13 (20.6)	<0.001
No	51 (43.9)		57 (49.1)		47 (40.5)	

Random blood glucose >200 mg/dL-*n* (%)						
Yes	54 (69.2)	0.001	38 (48.7)	0.92	20 (25.6)	0.051
No	45 (44.5)		50 (49.5)		40 (39.6)	

^1^WBC: white blood cells. ^2^Baseline D-dimer was measured in 48 (100%) patients in plasma group and 122 (93%) patients in control group.

**Table 3:** Multivariate analysis on factors confounding mortality.

	aOR^1^	95% CI	*p*-value
Convalescent plasma	0.45	0.12–1.6	0.22
Age	2.25	0.59–8.46	0.23
Hypertension	1.17	0.34–4.01	0.80
Obesity	14.1	1.25–166.7	0.032
Mechanical ventilation on admission	0.5	0.13–1.96	0.32
Mechanical ventilation during ICU stay	333	4.5–1,000	<0.001
WBC^2^ count	1.87	0.47–7.25	0.37
Neutrophil to lymphocyte ratio	7.32	1.82–29.4	0.005
PaO2 to FiO2 ratio	7.70	2.04–29.4	0.003
D-dimer	1.17	0.33–4.17	0.81
Creatinine serum	1.48	0.43–5.12	0.53
Random blood glucose	2.45	0.8–7.51	0.17

^1^Reference group of this regression modeling was length of stay of 7 days or more. ^2^WBC: White blood cells.

**Table 4:** Multivariate analysis on factors confounding prolonged ICU length of stay.

	aOR^1^	95% CI	*p*-value
Convalescent plasma	3.88	1.77–8.55	0.001
Age	1.32	0.62–2.78	0.467
Hypertension	2.14	1.05–4.35	0.036
Mechanical ventilation during ICU stay	1.55	0.77–3.15	0.219
WBC^2^ count	1.68	0.86–3.31	0.131
PaO2 to FiO2 ratio	1.81	3.27–4.24	0.173

^1^Reference group of this regression modeling was length of stay of 7 days or more. ^2^WBC: White blood cells.

**Table 5:** Multivariate analysis on factors confounding constant and deteriorating oxygen support device.

	aOR^1^	95% CI	*p*-value
Convalescent plasma	0.45	0.12–1.10	0.081
Obesity	9.18	2.0–42.1	0.004
Mechanical ventilation on admission	0.36	0.1–1.27	0.113
Mechanical ventilation during ICU stay	13.15	3.75–46.09	<0.001
WBC^2^ count	1.71	0.69–4.26	0.247
Neutrophil to lymphocyte ratio	2.5	1.07–5.85	0.034
PaO2 to FiO2 ratio	2.76	1.1–6.91	0.031
D-dimer	1.27	0.55–2.90	0.573
Creatinine serum	1.18	0.48–2.93	0.709
Random blood glucose	1.29	0.60–2.78	0.519

^1^Reference group of this regression modeling was patients with constant or deteriorated oxygen device requirements. ^2^WBC: White blood cells.

## Data Availability

The data of this study are available on request. Please mail author Nadya Ramadhania for further information on how to access the data.
